# Understanding the influence of processing conditions on the extraction of rhamnogalacturonan-I “hairy” pectin from sugar beet pulp

**DOI:** 10.1016/j.fochx.2019.100026

**Published:** 2019-05-03

**Authors:** Yujie Mao, Rui Lei, John Ryan, Fatima Arrutia Rodriguez, Bob Rastall, Afroditi Chatzifragkou, Charles Winkworth-Smith, Stephen E. Harding, Roger Ibbett, Eleanor Binner

**Affiliations:** a Department of Chemical and Environmental Engineering, Faculty of Engineering, University of Nottingham, University Park, NG7 2RD, UK; b Department of Food and Nutritional Sciences, University of Reading, PO Box 226, 13 Whiteknights, Reading RG6 6AP, UK; c National Centre for Macromolecular Hydrodynamics, School of Biosciences, University of Nottingham, Sutton Bonington LE12 5RD, UK; d School of Biosciences, University of Nottingham, Sutton Bonington LE12 5RD, UK

**Keywords:** Microwave-assisted extraction, Hydrothermal, Acid-free, Hairy pectin, Novel prebiotics, Rhamnogalacturonan-I, Sugar beet pulp

## Abstract

Sugar beet pectin is rich in rhamnogalacturonan-I (RG-I) region, which is a potential source of prebiotics. RG-I pectin cannot be extracted the same way as commercial homogalacturan-rich pectin using hot acid. Therefore, this study has explored several alternative methods, including microwave-assisted extraction (MAE) and conventional-solvent extraction (CSE) at atmospheric pressure using different solvents, and microwave-assisted hydrothermal extraction (MAHE) under pressure using water. No conclusive differences in microwave and conventional heating were found with heating rate controlled. The optimum treatment times of both MAE and CSE at 90 °C atmospheric pressure and regardless of the solvents used were 120 min; however, MAHE at 130 °C under pressure can dramatically reduce the time to 10 min. Alcohol-insoluble solids (AIS) extracted using pH13 solvent by MAE had both the highest RG-I yield at 25.3% and purity at 260.2 mg/g AIS, followed by AIS extracts using water by MAHE with 7.5% and 166.7 mg/g AIS respectively.

## Highlights

• Conventional and microwave-assisted extraction of “hairy” pectin from sugar beet.
• Determined effect of heating method, temperature, time & pH on yield & composition.
• No difference between microwave and conventional extraction under conditions tested.
• Strong alkaline is favoured in rhamnogalacturonan-I “hairy” pectin extraction.
• Hydrothermal water extraction can be an alternative to strong alkaline extraction.

## 1 Introduction

Pectins are complex heteropolysaccharides found in the primary cell wall and middle lamella of terrestrial plants. They consist of two major structural domains, homoglacturonan (HGA) and rhamnogalacturonoan-I (RG-I), which are covalently linked to one another (Ishii & Matsunaga, 2001). HGA is a linear homopolymer of α-1,4-linked-d-galacturonic acid (GalA) which can be methylesterified and acetylated to various degrees (Zhan, Janssen, & Mort, 1998) and is regarded as “smooth” pectin. The repeating unit of RG-I is the disaccharide 1,2-α-l-rhamnose-1,4-α-d-galacturonic acid. Many of the rhamnose residues have side chains containing linear and branched α-l-arabinofuranosyl (Araf), and/or β-d-galactopyranosyl (Galp) residues. Due to its highly branched nature, RG-I is often referred to as “hairy” pectin (Willats, McCartney, Mackie, & Knox, 2001). HGA-rich pectin are most widely used as hydrocolloids due to their ability to trap or bind water to form gels at low concentration (Ciriminna, Fidalgo, Delisi, Ilharco, & Pagliaro, 2016). RG-I-rich pectin is not suitable for this application as they do not gel; however, emerging research has demonstrated that RG-I-rich pectins with a high concentration of the side chains could be applied as potential sources of a new class of prebiotics, known as pectin-derived oligosaccharides (POS) (Babbar, Dejonghe, Gatti, Sforza, & Elst, 2016), which have superior ability to regulate microbiota in human intestine and anti-oxidant properties compared with other commercial oligosaccharides. Driven by the awareness of health, it has been projected that the global prebiotics market will reach US $4.8 billion by 2018 (Ciriminna et al., 2016).

Sugar beet pulp was chosen in this present study because of its prevalence in industry and promising RG-I pectin content. 7.5 million tonnes of sugar beet are grown each year in the UK (FAOSTAT, 2016). Sugar beet pulp is a co-product of the sugar refining industry, the majority of which is currently used for low value anaerobic digestion or animal feed. Sugar beet pulp after post-sugar extractions contains approximately 15–30% pectin (dry basis) (Adetunji, Adekunle, Orsat, & Raghavan, 2017), which has relatively low HGA and high concentration of RG-I region compared with commercial citrus peel or apple pectins that are rich in HGA (Gullón et al., 2013). Therefore, sugar beet pulp was selected as the model material in this study of RG-I pectin extractions.

RG-I pectin cannot be extracted the same way as commercial HGA-rich pectin, which uses a mineral acid at pH 1–3 and 80–90 °C (Ralet, Bonnin, & Thibault, 2002), because the hot acid results in the hydrolysis of neutral side chains of the RG-I region (Fraeye et al., 2007). This process also uses hazardous materials and produces significant volumes of acidic waste. Therefore, a clean, scaleable method of RG-I pectin extraction is required to enable the development of this new class of prebiotics, and over the last decades a variety of alternative extraction techniques have been explored.

Firstly, the extractions of pectin using other solvents have been analysed. Dilute alkaline solution was considered to be efficient to extract intact RG-I with high neutral side chains while it may destroy the HGA backbone (Khodaei, Karboune, & Orsat, 2016) and this could also result in massive alkaline waste. The use of chelating agents such as cyclohexanediaminetetraacetic acid (CDTA) or ethylenediaminetetraacetic acid (EDTA) can help the release of high methoxy pectin (HMP) from the cell wall through egg-box model and thus increase the pectin yield (Caffall and Mohnen, 2009, Renard and Thibault, 1993). However, chelating agents were shown to remain associated to HMP even after extensive dialysis (Renard & Thibault, 1993). Water as an environmentally benign solvent has also attracted attention, however the disadvantage of water in pectin extraction is its low efficiency in yields (Yeoh, Shi, & Langrish, 2008).

Secondly, apart from conventional heating, other heating methods have been explored, including microwave heating. Microwave-assisted extraction (MAE) is a novel extraction process that is widely reported to accelerate or enhance the solvent extraction of pectin (Adetunji et al., 2017). Microwaves can penetrate uniformly throughout the whole volume of material instantaneously and thus heat the material “volumetrically”, which allows microwave processes to be fast, continuous, compact and flexible in operation (Li, Fabiano-Tixier, Vian, & Chemat, 2013). Microwaves also heat “selectively”, which means that they heat different components of heterogeneous systems at different rates, and it is thought that this can lead to rupture of cells within biomass, resulting in higher extraction yields and the ability to treat recalcitrant materials (Adetunji et al., 2017, Chemat et al., 2009). Selective and volumetric heating also result in different temperature profiles compared with conventionally heated solvent-biomass systems, and enhanced microwave extraction rates have been attributed to the complementary heat and mass gradients (Chemat et al., 2009). The reduced extraction time of MAE are also thought to decrease pectin degradation (Fraeye et al., 2007). Another reported advantage is that MAE significantly lowers solvent requirements, which may result in running cost savings and reduced waste discharge (Adetunji et al., 2017).

Finally, hydrothermal treatment, carried out with hot compressed water, has been successfully employed for producing oligosaccharides from a variety of raw materials (Matharu, Houghton, Lucas-Torres, & Moreno, 2016). This methodology is favourable since 1) it is an environmentally friendly process as water is the only solvent; 2) corrosion problems can be avoided as no acid is added; 3) it is able to generate oligosaccharides in a single stage with promising yields and limited production of undesired sugar-degradation products; 4) it is a much faster reaction compared to other techniques (Martínez, Yáñez, Alonsó, & Parajó, 2010). There are several recent publications that coupled microwave heating with hydrothermal temperatures and have suggested the use of microwave-assisted hydrothermal extraction (MAHE) may help to minimise the side chain debranching and to maximise the pectin yields. For example, Matharu et al. (2016) found that using MAHE at 110 °C for 5 min and water as the solvent, they were able to extract up to 11.6 wt% pectin from mango peel, compared to 7.5 wt% using traditional acid extraction.

Although many studies have already explored one or several extraction techniques and conditions mentioned above, due to the many experimental variables that need to be controlled, meaningful comparison of different laboratory-scale set-ups is usually not possible. In particular, the control of heating rate in heating methods comparison was emphasised in the work conducted by Galan et al. (2017). To the best of our knowledge, there is no comprehensive study comparing all above techniques and conditions for RG-I pectin isolation in a systematic manner. The aim of this paper is therefore to understand the effect of processing conditions on the yield and purity of RG-I extracted from sugar beet pulp, with a view to informing process selection for the production of novel pectin-based products. Specifically, the objectives are to:

1. Compare the yield, purity and extraction time of RG-I pectin extracted using atmospheric pressure microwave and conventional solvent extraction (MAE and CSE respectively) using pH 1 to pH 13 and a chelator, controlling all other independent factors including stirring speed, vessel geometry, solid to liquid ratio (S/L) and heating rate.
2. Understand the effect of solvent choice (pH 1 – pH 13 and use of a chelator) on the yield and purity of RG-I pectin extracts.
3. Understand the effect of processing temperature and time on the yield and purity of RG-I extracted in water from 90 °C to 190 °C.

## 2 Materials and methods

### 2.1 Materials

Micronised sugar beet pulp powder (particle size range 20–200 μm, average approximately 90μm) was provided by Efficiency Technologies (Milton Keynes, UK). Sodium hexametaphosphate, hydrogen chloride (37% w/w HCl), sodium hydroxide (50% w/w NaOH) and isopropanol (IPA) were purchased from Sigma Aldrich (Dorset, UK).

### 2.2 MAE and CSE at atmospheric pressure using various types of solvents

The optimum solid to liquid ratio (S/L) was determined in scoping experiments (not presented here) to be 21.5 mL/g. Based on the maximum vessel size of the Miniflow SS200, all experiments at atmospheric pressure were therefore carried out using 43 mL extracting solvent added to 2 g of dry micronised sugar beet powder. Solutions used were: deionised (D.I.) water, a 1% (w/v) sodium hexametaphosphate chelator solution, dilute acid HCl solution at pH 1, 1.5 and 2 as well as dilute alkaline NaOH solution at pH 11, 12 and 13.

MAE was carried out using a Miniflow 200SS (Sairem, France) single mode microwave heating system at 2.45 GHz. A fibre optic temperature probe was inserted into the reaction vessel. A maximum incident power of 200 W was applied to the system to achieve a temperature set-point (90 °C). The reflected power was adjusted to zero before the treatment, so that the absorbed power can be as close as the incident power. CSE was carried out by adding the extraction vessel to a water bath preheated to the set temperature.

**Fig. 1:**
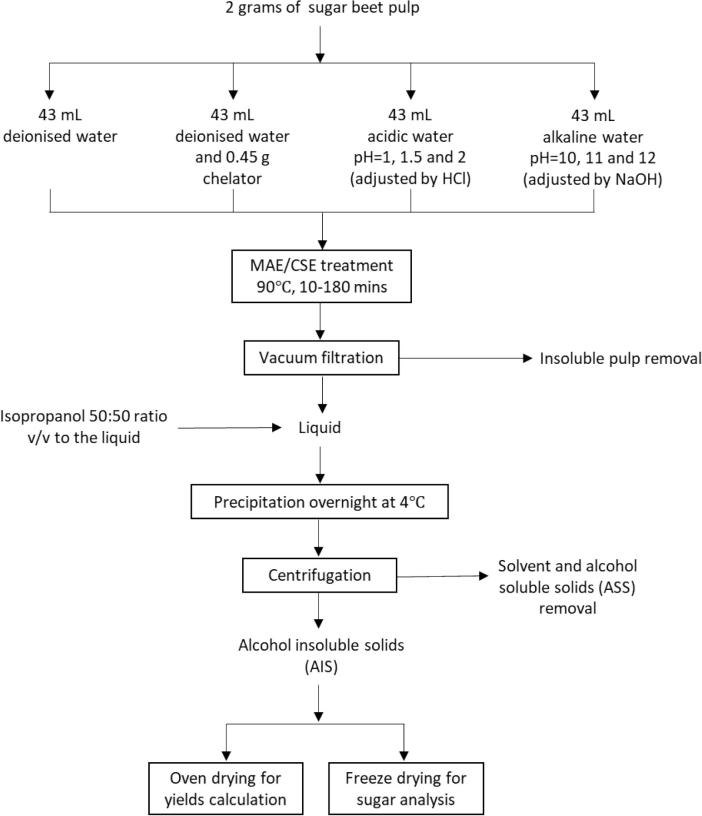
Schematic of the pectin extraction method at atmospheric pressure and 90 °C.

After heating, solid beet pulp residue was removed by filtration. The resulting liquid was centrifuged at 3900 rpm for 40 min and an equal volume of IPA was added to the supernatant for the precipitation of alcohol insoluble solid (AIS). Samples were stored overnight at 4 °C. Then the samples were centrifuged for another 40 min at 3900 rpm and the supernatant was discarded. The pellets were either oven-dried overnight at 60 °C to determine yield or freeze-dried for sugar analysis. All experiments were performed in triplicate. Fig. 1 shows a schematic of the extraction method.

### 2.3 MAHE under pressure using water

MAHE was carried out using a Monowave 200 microwave (Anton Parr, St Albans, UK). Although the Monowave vessels are smaller than those of Miniflow, for comparison at same S:L ratio of 21.5 mL/g, 0.8 g of dry sugar beet powder was added to 17.2 mL of D.I. water for Monowave experiments. A maximum power of 800 W was applied to the system, but there is no facility in the Monowave system to monitor reflected power, and so the absorbed power is unknown. The samples were heated to a desired temperature (90–190 °C) and held at this temperature for the required amount of time (0, 5, 10 and 15 min). Stirrer speed was set at 800 rpms. The same procedures, as described above, was then used to purify the sugar beet extracts.

**Fig. 2:**
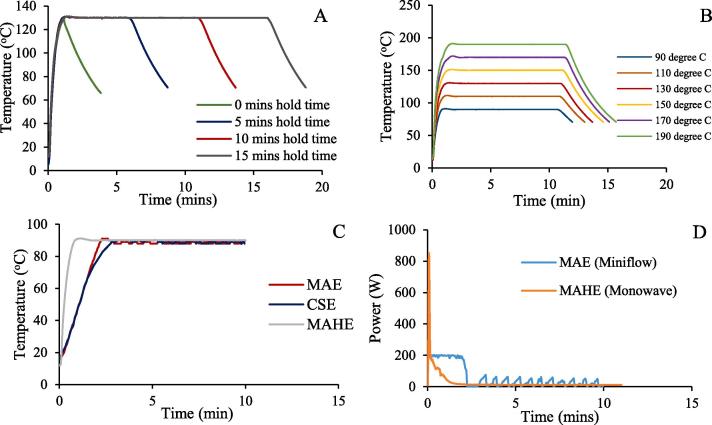
Heating curves for A. MAHE at 130 °C with holding times of 0, 5, 10 and 15 min; B. MAHE at 10 min hold time of 90, 110, 130, 150, 170 and 190 °C; C. MAE, CSE and MAHE at 90 °C; D. incident power profile of MAE at 90 °C 10 min using Miniflow and MAHE at 130 °C 10 min hold time using Monowave.

Fig. 2A and B show examples of the temperature profiles for the MAHE at various temperatures and hold times. Fig. 2C shows the heating rates of MAE, CSE and MAHE at 90 °C. Fig. 2D shows the incident power profile of MAE (Miniflow) and MAHE (Monowave). It needs to be noted that there is negligible difference between the heating rate of MAE and CSE; while MAHE has much higher heating rate, which makes the results from atmospheric pressure extractions not comparable to hydrothermal extractions even at same temperature 90 °C and treatment time 10 min. This higher heating rate in MAHE is due to the higher power rating and smaller volumes of sample input of the Monowave.

### 2.4 Yield and purity calculations

The extract quantity was determined by yields using the following equation:

(1) $$AIS\;Yield\left(\%\right)=\frac{Weight\;of\;oven\;dried\;alcohol\;insoluble\;solids(AIS)}{Weight\;of\;dry\;raw\;sugar\;beet\;pulp\;(RSB)}\times 100\%$$

AIS yields and error bars were calculated based on the average and standard deviation of triplicate runs. Varying only one variable a time, the optimum conditions of different extraction methods were found when their optimum AIS yields were reached.

The yields of RG-I pectin extracted was determined using the following equation (in other papers, RG-I yields (%) is also referred as RG-I region extractability (Kaya, Sousa, Crepeau, Sorensen, & Ralet, 2014)):

(2) $$RG-IYield\left(\%\right)=\frac{Percentage\;of\;RG-I\;region\;in\;AIS\;\times \;AIS\;Yield(\%)}{Percentage\;of\;RG\;I\;region\;in\;RSB}\times 100\%$$

where the percentage of RG-I region in AIS is also called RG-I purity (mg/g AIS), which is the sum of the sugar contents of rhamnose, arabinose and galactose (Rha + Ara + Gal) in AIS extracts.

### 2.5 Galacturonic acid (GalA) analysis

Soluble pectin extracts, lyophilised using a LyoDry freeze dryer (Mechatech Systems, Bristol, UK), were diluted to give an estimated concentration of 125 μg/mL in D.I water. Then the solutions were dispensed into pyrex boiling tubes, followed by the addition of 40 μL 4M potassium sulphamate and 2.5 mL of concentrated sulphuric acid. The mixture was heated to 99 °C for 20 min, which allowed the break-down of the HGA backbones into GalA. The solution was then cooled under running water. 80 μL of a solution of 0.15 wt% m-hydroxydiphenyl in 0.5 wt% NaOH was added into each tube and shaken on an orbital shaker to mix. The tubes were left to stand for 10 mins to allow a pink colour to develop. The absorbance of the pink solution was determined by UV–Vis chromatography (Jenway model 7315, Cole-Parmer Ltd UK) at a wavelength of 525 nm. Zero absorbance reference was set with D.I. water. The GalA standard solution was made at concentrations from 0 to 97 mg/L and was treated the same way as above. The results and error bars of GalA composition were determined by 4 repeats ((2 freeze-dried samples) × (duplication measurements of each sample in UV–Vis), such that the error bars include both the errors from extraction method and sugar analysis method).

### 2.6 Neutral sugar analysis

The freeze-dried pectin extract was hydrolysed with concentrated sulphuric acid using the same method as in the GalA assay. After the pre-hydrolysis, 100 μL of supernatant was added to 10 mL of 10 mM NaOH. 1 mL of the resulting solution was used for sugar analysis. High-performance anion exchange chromatography with pulsed amperometric detection (HPAEC-PAD) (Dionex, UK) using a CarboPac PA20 column and software Chromeleon was used. 10 mM NaOH (Solution A) was used as eluent and 200 mM NaOH (Solution B) as the mobile phase; retention gradient and time as −10 ∼ −5, 0.5 mL/min, 100% Solution A; −5 ∼ 14, 0.4 mL/min, 100% Solution B; stand-by, 0.1 mL/min. Mixtures of sugar standards (l-rhamnose, l-arabinose, d-galactose, d-glucose and d-xylose) at various concentrations (1–20 mg/L) were used as external standards for identification and quantification. The results and error bars of neutral sugar composition were determined by 4 repeats ((2 freeze-dried samples) × (duplication measurements of each sample in Dionex), in which way the error bars include both the errors from extraction methods and sugar analysis methods).

## 3 Results and discussion

### 3.1 Process optimisation

**Fig. 3:**
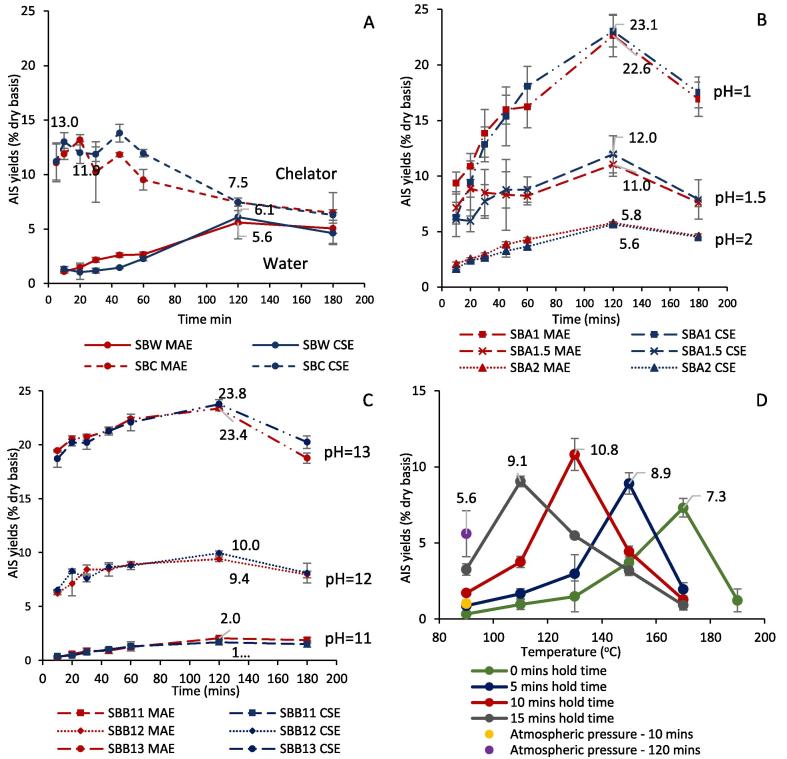
AIS Yields for A. sugar beet pectin atmospheric pressure extraction at 90 °C using water and 1% chelator (sodium hexametaphosphate) as the solvent. Where, SBW is sugar beet pectin extraction in water; SBC is sugar beet pectin extraction in chelator; B. Sugar beet pectin atmospheric pressure extraction 90 °C using dilute acid and alkaline at different pHs. Where SBA1/1.5/2 are sugar beet pectin extractions in dilute acid at pH = 1/1.5/2 correspondingly; C. Sugar beet pectin atmospheric pressure extraction 90 °C using dilute alkaline at different pHs. Where SBB11/12/13 are sugar beet pectin extractions in dilute alkaline at pH = 11/12/13 correspondingly; D. sugar beet pectin hydrothermal extraction using only water as the solvent.

The purpose of this paper is to understand the effect of a wide range of processing conditions on the yield and purity of RG-I extraction conditions from sugar beet pulp. We have as far as possible held all experimental conditions constant while changing only one variable at a time (solvent, processing time, temperature and heating method). To keep the size of the study manageable, the following experimental parameters (which could potentially affect outcomes) were not the focus of this study: stirrer speed, solid to liquid (S/L) ratio, microwave power and extraction vessel geometry. The first two were set using values determined in scoping experiments (not presented here) and not changed during any of the experiments. Unfortunately, it was not possible to hold the latter two (microwave power and vessel geometry) constant between the Miniflow (set temperature experiments) and Monowave (variable temperature experiments). This is because of differences in vessel geometry, electric field intensity (due to differences in microwave cavity design and power) and features of the two microwave power sources. The Miniflow was selected for the constant temperature experiments as the microwave power can be tuned (i.e. the proportion of the incident power that is absorbed by the sample can be maximised) and the incident and reflected power can be recorded, hence enabling the absorbed power to be calculated. In order to carry out experiments above 100 °C, however, the Monowave was required as it has the ability to monitor the temperature of samples under pressure. This has a higher maximum incident power but no facility to measure the reflected power or adjust the tuning, and the extraction vessels are smaller. Therefore, the latter two “set” variables (absorbed power and vessel geometry) were different between the two different sets of experiments. However, comparing the results for the Miniflow (90 °C) with water as the solvent (on Fig. 3A and the points marked “atmospheric pressure” on Fig. 3D) with the 90 °C Monowave points on Fig. 3D, the effect of the different microwave powers (or electric field intensities) and vessel geometries appear to be insignificant compared with the effect of other variables such as temperature and solvent.

The first optimisation step (presented in Fig. 3 A, B and C and discussed in more detail in Section 3.2) was to determine the effect of solvent selection and processing time. For these experiments, the temperature was set to 90 °C, and the microwave power was set to 200 W. The reason for this is that 200 W is the maximum power for the Miniflow apparatus used, and if there was any difference in extraction between conventional heating and microwave heating (resulting from the ability of microwaves to selectively heat different parts of the biomass-solvent system), we would expect this difference to be most pronounced at higher power densities, which are proportional to the electric field squared, which is a function of the microwave incident power.

### 3.2 AIS yields

#### 3.2.1 AIS yields for MAE and CSE at atmospheric pressure

The yields of AIS as defined in Eq. (1) were presented for atmospheric pressure MAE and CSE in Fig. 3A (water and chelator solvents), Fig. 3B (acidic solvents) and Fig. 3C (alkaline solvents). The AIS yields attained varied substantially across the experimental conditions tested, with optimal AIS yields varying between 2 and 24% depending on the solvent used.

##### 3.2.1.1 Comparison between microwave and conventional heating

In all cases, there was no conclusive difference between the maximum AIS yields achieved by MAE and CSE, nor the time to achieve maximum extraction. This is contrary to results presented in many publications, which indicated that microwave heating can dramatically reduce extraction time and increase yield, but most of the work comparing microwave and conventional heating were not performed controlling all variables apart from heating methods. For example, Bagherian, Ashtiani, Fouladitajar, and Mohtashamy (2011) reported that the optimal AIS yield of grapefruit pectin extraction was 26.3% using microwave heating at 900 W for 2 min, compared with 19.2% using conventional extraction for 90 min and 90 °C (based on only one treatment condition), where in the comparison the treatment temperature and heating rate were not controlled to be the same. It is worth noting, however, that we are only aware of two other publications that have decoupled the effects of selective and volumetric heating by using the same small extraction vessel volume to achieve a comparable bulk heating rate when comparing conventional and microwave heating (Calinescu et al., 2017, Galan et al., 2017). Both of these papers related to the extraction of polyphenols from seabuckthorn leaves using a 50% ethanol/50% water solvent, and in both cases the time to maximum extraction was observed to be comparable for MAE and CSE. Both papers also noted that the maximum yield of target extract (as measured by Gallic acid equivalent, GAE/g plant material) was moderately higher for MAE that CSE (e.g. 163 and 150 mg GAE/g respectively (Galan et al., 2017)). By using the same bulk heating rate, the effect of microwave volumetric heating is essentially negated. Of course the ability to heat volumetrically may pose some advantages in scale-up (e.g. energy savings and space saving), but this is not straightforward and was discussed in the above mentioned paper (Galan et al., 2017). The increased yields observed in the extraction of polyphenols from seabuckthorn leaves were therefore attributed to the ability of microwaves to selectively heat the biomass (Galan et al., 2017); however, this effect was not observed here in the extraction of AIS from sugar beet pulp. It is noted that there could be various reasons for this including the different physical and chemical characteristics of the target extracts, their location within the plant cells (inter- versus intracellular), the different solvents used (water versus ethanol/water) and feedstock characteristics and sample preparation method (micronised sugar beet pulp (approx. 90μm) versus <0.5 mm ground leaves). It is also noted that this only applies to the electric fields applied in the Miniflow cavity used in these experiments; it is possible that higher electric fields may lead to different results. Finally, for the results presented here for solvents with pH 1–7, there may be a slight advantage of microwave processing for extraction times up to 30 min, but the results are inconclusive. Understanding of the effects of these many variables on extraction performance is the subject of ongoing research in our group.

##### 3.2.1.2 Comparison between various solvents used

For sugar beet atmospheric pressure extractions using water, in Fig. 3A, the optimum AIS yields of 5.6% and 6.1% for MAE and CSE extractions respectively were both found at 120 min; however, after 120 min, AIS yields decreased, which may be due to β-elimination of the pectin (Fraeye et al., 2007), where the molecular weight of the pectin decreased to a level where the polysaccharide would not be precipitated in alcohol. Many authors have found similar results where yield initially increases with time and/or temperature, but after reaching a maximum, the yield drops rapidly (Adetunji et al., 2017). Using a chelator, the AIS yield was almost double that of the water extractions, and the maximum AIS yield was achieved in just 10 min for 11.9% and 13.0% for MAE and CSE extractions respectively. After 10 min the AIS yield decreased, which again may be due to β-elimination of the pectin.

For sugar beet atmospheric pressure extractions under acidic conditions, in Fig. 3B, all the optimum AIS yields for both MAE and CSE were found at 120 min. Lowering the pH increased AIS yields. Optimum AIS yields of 22.6% and 23.1% for MAE and CSE extractions respectively were reported at pH = 1; 11.0% and 12.0% respectively at pH = 1.5; 5.8% and 5.6% respectively at pH = 2. This is because more acidic condition helps to open the cell wall structure, which leads to better solubility of pectin into the extracting solvents and thus better yields (Adetunji et al., 2017). After 120 mins, AIS yields at all acidic pHs started to decrease, however the lower the pH, the faster the degradation rate. This is because at a pH lower than 2, acid hydrolysis dominates pectin degradation, and this effect increases with decreasing pH (Fraeye et al., 2007).

For the sugar beet atmospheric pressure extractions under alkaline conditions, shown in Fig. 3C, again all the optimum AIS yields for both MAE and CSE were found at 120 min. Increasing the pH increased AIS yields. Optimum AIS yields of 23.4% and 23.8% for MAE and CSE extractions respectively were reported at pH = 13; 9.4% and 10.0% respectively at pH = 12; 2.0% and 1.7% respectively at pH = 11. This is because alkaline conditions can break the bonds between the pectin and the cell wall in a similar manner to acidic solvents and thus increasing alkalinity leads to higher AIS yields (Yeoh et al., 2008). After 120 min, AIS yields at all alkaline pHs started to decrease, and this effect was accelerated by increasing pH. This is because at a pH from neutral to alkaline, methoxylation dominates pectin degradation and the higher the pH the stronger the methoxylation (Fraeye et al., 2007). It needs to be noted that the AIS yields achieved at pH = 11 were much lower than those at pH = 7. This is because although pH = 11 alkaline solvent was added, the pH of solvent and solid mixture was 5.5, which still represented an acidic condition. While addition of pH = 7 (D.I. water) solvent to the solid made the mixture reached pH = 4.8. In other words, the comparison of AIS yields under pH = 11 and 7 solvent condition is actually the comparison of two acidic pHs. Therefore, pH = 7 solvent corresponding to a lower acidic pH can achieve better AIS yields than that of pH = 11 solvent.

It is finally noted that, with the exception of the chelator experiments, the optimal extraction time for all experiments was somewhere between 60 and 180 min (with the maximum measured AIS yield at 120 min). The difference in extraction characteristics when using the chelator was better understood after characterisation of the AIS extracts, and so is discussed further in Section 3.2.2.

#### 3.2.2 AIS yields for MAHE under pressure

Fig. 3D shows the AIS yields for MAHE using only water as the solvent for hold times of 0–15 min and temperatures ranging from 90 to 190 °C. These experiments were carried out in a different microwave cavity (Monowave) than the atmospheric pressure MAE experiments (Miniflow) (see 2.2, 2.3), and therefore the incident and reflected power and vessel geometry were different, leading to different heating rates (see Fig. 2). Therefore, AIS yields for hold times of 10 and 120 min carried out in the Miniflow are also reported in Fig. 3D for comparison. This comparison shows that the Monowave gives slightly higher AIS yields than the Miniflow for the same hold time and temperature. This is attributed to the faster heating rate of the Monowave leading to less pectin degradation. Nevertheless, the AIS yields are close enough to conclude that higher AIS yields are achievable for all hydrothermal processing temperatures (i.e. >100 °C) compared to atmospheric pressure MAE at 90 °C.

Fig. 3D shows that for each hold time there is an optimal processing temperature, above which AIS yield drops off sharply. Longer the hold time, lower the optimum temperature was required. Reducing the hold time to 0 min maximises this optimal temperature at 170 °C, yielding 7.3% AIS. The highest AIS yield, however, was achieved at 130 °C and 10 min; this was 10.8%, nearly double the maximum AIS yield of 5.6% achieved at MAE 90 °C atmospheric pressure with a 2 h extraction time. Moreover, longer the hold time from 0 to 10 mins, higher the optimum AIS yields; however optimum AIS yield started to decrease to 9.1% when further increase the treatment time to 15 min. A combination of the following factors is the likely reason for the large increase in MAHE AIS yields. Firstly, the dielectric constant of hot pressurised water (or subcritical water) is lower than water at room temperature, which changes its solvent properties, i.e. it becomes less polar (Ruiz, Rodriguez-Jasso, Fernandes, Vicente, & Teixeira, 2013). This could potentially lead to a larger difference in dielectric properties between the solvent water and the sugar beet pulp, which enhances selective heating of sugar beet pulp. Secondly, it becomes easier for hot pressurised water to penetrate into plant cell wall, which leads to the opening of cell wall structure and better dissolution of pectins (Ruiz et al., 2013). Moreover, dissociation of water at high temperatures results in a lowering of its pH and thus gives water similar properties as dilute acid (Plaza & Turner, 2015).

As stated above, for each hold time the yield decreased sharply when the temperature was increased above the optimal point. This correlated with visible degradation of the extract; at 190 °C for 0 min hold time and 170 °C for 5, 10 and 15 min, the extracted AIS was in black colour and had a caramelised odour. This demonstrates that higher temperatures can quickly release pectin from the cell wall structure but when it is in solution will rapidly degrade. In this case, microwave heating shows its advantage when applying hydrothermal extractions as microwave allows quicker heating.

#### 3.2.3 Degradation of pectin

During thermal processing, pectins are subjected to depolymerisation and demethoxylation reactions. Acid hydrolysis and β-elimination are two major mechanisms of depolymerisation depending on the process, pH and degree of methylation (DM) of the pectin (Fraeye et al., 2007). β-elimination proceeds in uronic acids on the glycosidic bonds and the prerequisite is the presence of a methyl ester group at the C-6 position (Keijbets & Pilnik, 1974). Demethoxylation can take place during the process and may impact β-elimination (Kravtchenko, Arnould, Voragen, & Pilnik, 1992). It is believed that β-elimination and demethoxylation are two competitive reactions (De Roeck et al., 2009). The parameters influencing pectin degradation include elevated temperature, pH, DM and the presence of calcium ions. Detailed influences of each parameter are summarised below in Table 1.

**Table 1:** Summary of pectin degradation mechanisms during thermal processing. Summarised from De Roeck et al., 2009, Fraeye et al., 2007, Kravtchenko et al., 1992, Keijbets and Pilnik, 1974.

Factors	Relevant extraction solvents	Dominant degradation mechanism	
High temperature	All solvents	Both beta-elimination and methoxylation accelerates with increasing temperatures	
pH	Acid and base solvents	At pH below or around 2	Acid hydrolysis
		At pH 3–7	Beta-elimination
		At neutral to alkaline condition	Methoxylation
Degree of methylation (DM)	Acid and base solvents	At acidic condition around 3	Acid hydrolysis increases with decrease DM
		At neutral to alkaline condition	Beta-elimination could be retarded when DM is below a certain level
Treatment time	All solvents	More degradation happens in longer treatment time, but the mechanisms vary between other extraction parameters (temperature, pH, DM etc.)	
Calcium ions	Chelator solvent	Retention of cell wall firmness by the addition of calcium ions is thought to be related to the ability of divalent cations to bind the pectic matrix with ‘egg-box’ cross-links	

It should be noted that the above pectin degradation mechanisms were primarily studied on HGA pectin. More work needs to be done to fully understand the impact of thermal processing on the degradation of RG-I pectin side chains.

### 3.3 Chemical compositions

The optimal AIS yield points (120 min for all atmospheric pressure extractions; 10 min for hydrothermal extraction using water; 10 min for all atmospheric pressure extraction using chelator) were selected for sugar analysis. Results are listed in Table 2A and B. As with the extraction AIS yields, the mono-sugar contents again shows no conclusive difference between microwave and conventional heating at atmospheric pressure and 90 °C, which was also due to the negligible difference in heating rate. Therefore, in the discussion below only MAE results are shown but the same applies to CSE. The mono-sugar contents show great variation when using various solvents.

**Table 2:** Chemical compositions of untreated sugar beet pulp and sugar beet extracts. Where, GalA is galacturonic acid; Rha is rhamnose; Ara is arabinose; Gal is galactose; Glu is glucose; Xyl is xylose; Total sugars = GalA + Rha + Ara + Gal + Glu + Xyl; RG-I region = Rha + Ara + Gal; Sugar ratio 1 = GalA/(Rha + Ara + Gal + Glu + Xyl), which represents the linearity of pectin; Sugar ratio 2 = Rha/GalA, which represents the contribution of RG to pectin population; Sugar ratio 3 = (Ara + Gal)/Rha, which represents the branching of RG-I. Sugar ratio definitions are based on Houben, Jolie, Fraeye, Van Loey, and Hendrickx (2011).

			AIS yields	GalA.	Rha.	Ara.	Gal.	Glu.	Xyl.	Total sugars	RG-I region purity	RG-I yields	Sugar ratio		
			% dry basis	mg/g RSB	mg/g RSB	mg/g RSB	mg/g RSB	mg/g RSB	mg/g RSB	mg/g RSB	mg/g RSB	% dry basis	1	2	3
Untreated sugar beet pulp			/	480.7 ± 1.2	4.0 ± 0.1	186.4 ± 2.1	49.6 ± 0.9	241.3 ± 1.8	19.6 ± 0.7	981.7 ± 6.8	240.0 ± 3.1	/	1.0 ± 0.1	0.008 ± 0.001	58.6 ± 0.6
Solvent	Time	Extraction method	AIS yields	GalA.	Rha.	Ara.	Gal.	Glu.	Xyl.	Total sugars	RG-I pectin purity	RG-I yields	Sugar ratio		
	min		% dry basis	mg/g AIS	mg/g AIS	mg/g AIS	mg/g AIS	mg/g AIS	mg/g AIS	mg/g AIS	mg/g AIS	% dry basis	1	2	3
Chelator	10	MAE 90 °C	11.0 ± 0.5	277.7 ± 2.1	1.1 ± 0.1	16.1 ± 0.1	11.3 ± 0.1	28.5 ± 1.1	0.5 ± 0.2	321.3 ± 3.7	28.5 ± 0.3	1.4 ± 0.1	6.4 ± 0.6	0.004 ± 0.001	24.0 ± 0.4
Chelator	10	CSE 90 °C	13.0 ± 0.9	276.5 ± 1.5	2.0 ± 0.0	17.4 ± 0.1	12.1 ± 0.1	31.5 ± 0.6	0.6 ± 0.1	323.1 ± 2.4	31.5 ± 0.2	1.7 ± 0.1	5.9 ± 0.4	0.007 ± 0.000	15.0 ± 1.2
Chelator	120	MAE 90 °C	7.5 ± 0.1	465.4 ± 1.0	2.7 ± 0.6	47.1 ± 0.1	18.0 ± 0.5	67.8 ± 0.8	2.9 ± 0.3	603.4 ± 3.3	67.8 ± 1.2	2.1 ± 0.2	3.4 ± 0.7	0.006 ± 0.001	24.1 ± 0.9
Chelator	120	CSE 90 °C	7.5 ± 0.4	419.9 ± 0.8	2.9 ± 0.4	44.7 ± 0.6	17.3 ± 0.1	65.0 ± 1.5	2.8 ± 0.2	553.4 ± 3.6	65.0 ± 1.1	2.0 ± 0.1	3.2 ± 0.5	0.007 ± 0.001	21.3 ± 1.3
pH = 1	120	MAE 90 °C	22.6 ± 1.9	859.9 ± 2.3	7.4 ± 0.1	57.2 ± 0.1	20.7 ± 0.1	85.3 ± 1.2	2.2 ± 0.2	990.1 ± 4.0	85.3 ± 0.3	8.1 ± 0.2	6.6 ± 0.0	0.009 ± 0.001	10.5 ± 0.4
pH = 1	120	CSE 90 °C	23.1 ± 1.4	860.4 ± 2.1	7.5 ± 0.8	56.2 ± 0.4	19.5 ± 0.2	83.2 ± 2.1	2.4 ± 0.2	990.7 ± 5.8	83.2 ± 1.4	8.0 ± 0.2	6.6 ± 0.1	0.009 ± 0.000	10.2 ± 0.2
pH = 1.5	120	MAE 90 °C	11.0 ± 1.1	820.9 ± 0.5	7.8 ± 0.0	77.7 ± 0.3	25.3 ± 0.1	110.9 ± 1.5	1.8 ± 0.4	966.3 ± 2.8	110.9 ± 0.4	5.1 ± 0.1	5.7 ± 0.5	0.01 ± 0.000	13.2 ± 0.3
pH = 1.5	120	CSE 90 °C	12.0 ± 1.7	821.3 ± 0.6	7.8 ± 0.6	75.1 ± 0.1	27.4 ± 0.8	110.4 ± 0.5	2.0 ± 0.0	967.0 ± 2.6	110.8 ± 1.5	5.5 ± 0.2	5.6 ± 0.5	0.010 ± 0.001	13.1 ± 0.7
pH = 2	120	MAE 90 °C	5.8 ± 0.1	799.8 ± 1.5	8.7 ± 0.3	93.0 ± 0.0	39.2 ± 0.1	140.9 ± 1.8	1.1 ± 0.0	970.4 ± 3.7	140.9 ± 0.4	3.4 ± 0.1	4.7 ± 0.1	0.011 ± 0.000	15.3 ± 0.1
pH = 2	120	CSE 90 °C	5.6 ± 0.2	794.3 ± 1.4	8.5 ± 0.2	93.7 ± 0.2	40.9 ± 0.9	143.1 ± 1.8	1.9 ± 0.3	965.9 ± 4.8	143.1 ± 1.3	3.4 ± 0.1	4.6 ± 0.2	0.011 ± 0.000	15.9 ± 0.8
pH = 7 (Water)	120	MAE 90 °C	5.6 ± 1.5	795.8 ± 2.6	8.8 ± 0.0	98.4 ± 0.3	39.2 ± 0.3	146.5 ± 1.5	1.4 ± 0.1	954.7 ± 4.8	146.5 ± 0.6	3.4 ± 0.1	5.0 ± 0.1	0.011 ± 0.000	15.7 ± 0.0
pH = 7 (Water)	120	CSE 90 °C	6.1 ± 0.6	793.0 ± 0.1	8.3 ± 0.9	98.3 ± 0.9	40.3 ± 0.3	146.9 ± 1.2	1.5 ± 0.4	953.8 ± 3.8	146.9 ± 2.1	3.7 ± 0.2	4.9 ± 0.3	0.010 ± 0.000	16.7 ± 0.1
pH = 7 (Water)	10	MAHE 130 °C	10.8 ± 1.1	790.3 ± 0.7	9.3 ± 0.7	110.8 ± 0.1	46.6 ± 0.9	166.7 ± 0.7	1.5 ± 0.0	968.6 ± 3.1	166.7 ± 1.7	7.5 ± 0.4	4.4 ± 0.3	0.012 ± 0.001	17.0 ± 0.2
pH = 11	120	MAE 90 °C	2.0 ± 0.1	706.4 ± 0.9	9.4 ± 0.4	102.8 ± 0.9	41.3 ± 0.7	153.5 ± 0.9	1.3 ± 0.3	885.4 ± 4.1	153.5 ± 2.0	1.1 ± 0.0	4.0 ± 0.5	0.013 ± 0.001	15.3 ± 0.4
pH = 11	120	CSE 90 °C	1.7 ± 0.2	705.7 ± 0.7	9.0 ± 0.1	102.0 ± 0.2	43.9 ± 1.1	154.9 ± 2.3	1.8 ± 0.1	891.7 ± 4.5	154.9 ± 1.4	1.3 ± 0.0	3.8 ± 0.2	0.013 ± 0.000	16.2 ± 0.7
pH = 12	120	MAE 90 °C	9.4 ± 0.2	520.6 ± 1.9	12.7 ± 0.9	108.5 ± 0.6	53.6 ± 0.5	174.7 ± 1.7	2.5 ± 0.4	738.3 ± 5.0	174.7 ± 2.0	6.8 ± 0.2	2.4 ± 0.4	0.024 ± 0.001	12.8 ± 0.2
pH = 12	120	CSE 90 °C	10.0 ± 0.2	524.0 ± 0.4	12.1 ± 0.7	108.7 ± 0.7	50.2 ± 0.9	170.9 ± 2.4	2.7 ± 0.1	741.7 ± 5.2	170.9 ± 2.3	7.1 ± 0.3	2.4 ± 0.1	0.023 ± 0.000	13.2 ± 0.5
pH = 13	120	MAE 90 °C	23.4 ± 0.2	280.3 ± 0.6	21.0 ± 1.1	159.4 ± 0.7	79.9 ± 0.6	260.2 ± 1.1	3.3 ± 0.2	600.9 ± 4.3	260.2 ± 2.4	25.3 ± 1.8	0.9 ± 0.3	0.075 ± 0.001	11.4 ± 0.0
pH = 13	120	CSE 90 °C	23.7 ± 0.4	284.4 ± 1.1	21.1 ± 0.2	155.3 ± 0.9	77.8 ± 0.5	254.1 ± 0.2	3.2 ± 0.4	602.5 ± 3.3	254.1 ± 1.6	25.2 ± 1.2	0.9 ± 0.1	0.074 ± 0.001	11.1 ± 0.4

#### 3.3.1 The use of water

Water was used as the solvent for both atmospheric pressure extractions and hydrothermal extractions. The optimal AIS yields of hydrothermal extraction was 10.8% achieved at 10 min treatment time and 130 °C compared to atmospheric pressure extraction AIS yields 5.6% for MAE at 120 min and 90 °C; however, the chemical compositions are very similar between atmospheric pressure and hydrothermal extractions. This indicates that the extract chemical composition is dominantly influenced by different solvent natures rather than extraction temperature and pressure; while extract AIS yields can be influenced by solvent as well as temperature and pressure. Although as mentioned above at hydrothermal condition, the property of water might become more like dilute acid, the chemical composition of hydrothermal water extracts are not very similar to atmospheric acid extracts. This is because the change of water pH in hydrothermal process is minor (for example, around 5.7 at 150 °C (Plaza & Turner, 2015)), which did not represent a great change compared with dropping it to pH 2. Also the increase of AIS yields in hydrothermal is due to a combination of many aspects not only lowering pH.

#### 3.3.2 The use of chelator

The extraction AIS yield using a chelator as the solvent at 10 min was double that of water at 120 min; however, the sugar analysis results showed that the total sugar content of using chelator as the solvent for 10 min were only 321.3 mg/g AIS and for 120 min were 603.4 mg/g AIS, which were both much lower than that of using water 954.7 mg/g AIS. This suggests that the better AIS yields achieved using chelator as the solvent may not be due to more pectin extracted but chelator itself remaining in the extract. This same issue was also discovered by Thibault, Renard, Axelos, Roger, and Crépeau (1993), who reported that chelator remained to associate with pectins even after extensive dialysis. Therefore, it might be concluded that using chelator as the solvent in the extraction of pectin is not of necessity.

#### 3.3.3 The use of acid and alkaline solvents

GalA is the main component of pectin HGA region, which is a straight backbone. In terms of GalA compositions, the lower the pH the higher the GalA content. The highest GalA content was 859.9 mg/g AIS for MAE using pH = 1 solvent. Increasing the GalA content also means the increase of the pectin linearity. The sugar ratio 1 that represents linearity of pectin reaches 6.6 at solvent pH = 1 compared to 0.9 at solvent pH = 13. This is because acid often promotes the extraction of GalA while alkali degrades the GalA (Fraeye et al., 2007). Similar results were reported by Kaya et al. (2014) in the extraction of citrus pectin; at pH = 1.6 the GalA extractability was approximately 95% and at pH = 4.6 the GalA extractability reduced to approximately 65%., where GalA extractability is the percentage of GalA extracted compared with total extractable GalA in the feedstock, and is analogous to Fig. 2 for RG-I Yield.

Rha is the main component in RG-I region backbone, connecting to RG-I neutral side chains that are primarily made of Ara and Gal. In terms of pectin neutral sugar contents, the lower the pH, the higher the Rha, Ara and Gal contents, with the most RG-I region extracted being 260.2 mg/g AIS for MAE achieved using pH = 13 solvent. Furthermore, sugar ratio 2, which represents the contribution of the RG region of the whole pectin population increased with increasing pH. It reached 0.075 at solvent pH = 13 compared to 0.009 at pH = 1. It is because acid attacks the pectin neutral side chains while on the other hand, alkaline conditions often preserve the side chains (Byg et al., 2012). Moreover, although Rha, Ara and Gal contents all generally increased with higher pH, the influences of pH on different side chain neutral sugars were slightly different. This can be illustrated by sugar ratio 3, which represents the branching of RG-I. At acidic pH the sugar ratio 3 value increased with increasing pH, maximised at 15.7 at neutral pH, then decreased with increasing pH in the alkaline region. This implies that using water as the solvent, the RG hairy region of extracted pectin may have more congregated side chains attached to each Rha residue.

Higher alkaline pH was also found to result in extracts with higher Glu and Xyl contents. This is because Glu and Xyl are mainly from hemicellulose that is insoluble in water but soluble in alkaline solutions and higher the alkaline condition better the hemicellulose extraction (Doner & Hicks, 1997). Those hemicellulose fractions were not purified after AIS precipitation.

### 3.4 Comparisons of different RG-I pectin extraction methods

**Fig. 4:**
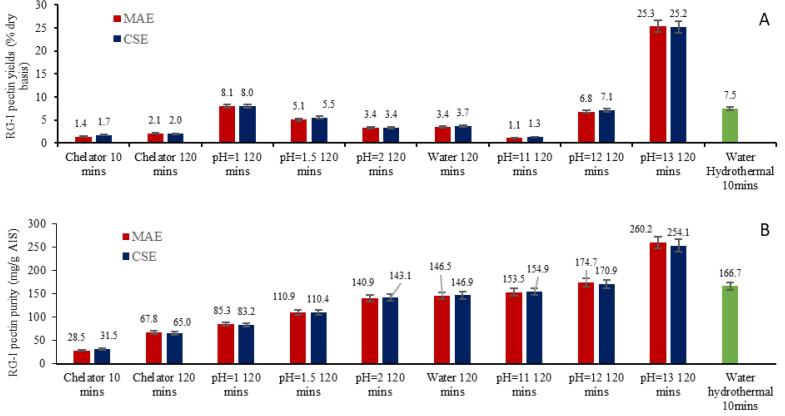
RG-I extraction using different extraction methods A. RG-I pectin yields (% dry basis). B. RG-I pectin purity (mg/g AIS).

Different RG-I pectin extraction methods are compared depending on the yields and the purity of the RG-I pectin extracted. RG-I pectin yield represents how much RG-I pectin could be extracted from raw sugar beet pulp and it was calculated based on Eq. (2) and shown below in Fig. 4A. RG-I pectin purity represents how many grams of RG-I region was present in each gram of AIS extracted, and it is shown below in Fig. 4B and Table 2B.

As discussed in Section 3.2.3, RG-I pectin purity increases with the increase of extracting solvent pH because acid destroys the RG-I region, while alkali helps to preserve it. The changes of RG-I pectin yields are more complex as they depend on both the yields of total AIS extracted and the RG-I pectin purity in the AIS. For example, although the pH = 1 acid extract has the second highest RG-I pectin yield of 8.1% by MAE, it has the lowest RG-I purity; in contrast, the pH = 13 alkaline extract has both highest RG-I pectin purity at 260.2 mg/g AIS and highest yields at 25.3% by MAE. Under hydrothermal conditions, extract obtained using water at 130 °C and 10 min also has relatively high RG-I pectin yield at 7.5%, which is similar to those of pH = 1 acid extract and pH = 12 alkaline extract; water hydrothermal extract also has relatively high RG-I pectin purity at 166.7 mg/g AIS, which is similar to pH = 12 alkaline extract. The yield and purity of RG-I extracts are similar using water hydrothermal and pH = 12 alkaline atmospheric method. Extracts by other solvents at atmospheric pressure are low either in RG-I pectin yields, RG-I pectin purity or both. With this knowledge, this study therefore provides key insights into the selection of processes to extract RG-I pectin. pH = 13 alkaline at atmospheric pressure is apparently the best option based on yield and purity, followed by pH = 12 alkaline at atmospheric pressure and water under hydrothermal processing conditions.

However, constraints including process safety concerns, environmental impact and cycle time also need to be taken into account. Firstly, using strong alkaline solvents can cause danger in process operation as well as posing waste disposal issues and pollution if not treated properly. The use of strong alkaline conditions may also require a further purification/neutralisation step. However, water is a cheap, environmentally friendly and food-grade solvent, which does not require further treatment before disposal. All atmospheric pressure extractions were conducted at 90 °C; while hydrothermal extractions would be under a temperature of 130 °C and a vapour pressure of 2.7 bar. Atmospheric pressure extractions can be considered safer, but hydrothermal of 2.7 bar is not very high in the context of industrial processes. For example, a common industrial process of nitric acid production has operating pressure in the range of 1.7–13 bar (Martín, 2016). Secondly, as for cycle time, MAHE showed an obvious advantage that it only took 10 min at 130 °C to reach the optimal extraction condition rather than 120 min at 90 °C using atmospheric pressure extractions. It may also be possible to achieve 0 min treatment time using hydrothermal extractions, however, further work needs to be completed to understand pectin extraction and degradation kinetics to achieve this. If 0 min can be applied, it may dramatically reduce the process cycle time, enabling a continuous system design. Any other related industrial constrains (e.g. economics) also require further study.

It worth noting that as different raw materials have huge pectin content variations and structural diversity (Muller-Maatsch et al., 2016), the effect of feedstock characteristics is also of significance in order to propose the best pectin and/or RG-I pectin extraction method. This was not studied in this work.

## 4 Conclusion

In this work, the influences of different extraction techniques and conditions on the yield and purity of total AIS and RG-I recovered from sugar beet pulp were analysed in a systematic manner, with the aim of informing the design of future RG-I pectin extraction processes. Those extraction techniques were atmospheric pressure microwave-assisted, conventional solvent extraction (MAE and CSE respectively) as well as pressurised microwave-assisted hydrothermal extraction (MAHE). The extraction conditions studied were different solvents (pH 1–pH 13 and use of a chelator), treatment time and processing temperatures. In order to achieve meaningful comparison of different experiment set-ups, all other independent factors including stirring speed, vessel geometry, solid to liquid ratio (S/L) and most importantly heating rate were controlled.

No conclusive difference in AIS yield and quality between microwave and conventional heating were observed. This is because the effects of microwave volumetric heating here nullified by using similar heating rates for the conventional and microwave heating experiments, and also indicates that in the case of micronised sugar beet pulp, there are no discernible microwave selective heating advantages for the electric field intensities achieved in these experiments. This is not to say that MAE will not pose an advantage in every extraction.

Optimum AIS yields were achieved at 120 min for all solvents in atmospheric pressure. At reaction times greater than 120 min AIS yields decreased due to pectin degradation. Using a chelator as the solvent achieved much better AIS yields but the chemical composition analysis showed that the better yields may not be due to more pectin extracted but chelator itself remaining in the extract, which suggested that chelator may not be suitable for pectin extraction. Low pH favoured HGA region extraction; at pH = 1, the AIS yields were 22.6% for MAE extractions, giving the highest GalA contents of 859.9 mg/g AIS, although it degraded RG-I side chains. High pHs also lead to high AIS yields, and alkaline conditions helped to preserve the neutral side chains; at pH = 13, the yields were 23.4% with total hairy pectin contents of 260.2 mg/g AIS. MAHE at 130 °C in a much shorter treatment time of 10 min using only the green solvent water can achieve both good AIS yield 10.8% and hairy pectin content 166.7 mg/g AIS in extracting sugar beet pectin. Although rapid pectin degradation may occur in high temperatures, microwave allows quicker heating.

RG-I pectin recovery was assessed using RG-I pectin yield (how much RG-I pectin could be extracted from raw sugar beet pulp) and RG-I pectin purity (how many grams of RG-I region was present in each gram of AIS extracted). pH = 13 alkaline extracts had both the highest RG-I pectin purity at 260.2 mg/g AIS and the highest yields at 25.3% by MAE. Using pressurised MAHE, extracts obtained by using water at 130 °C and 10 min also had relatively high RG-I pectin yields at 7.5% and purity at 166.7 mg/g AIS, which was similar to pH = 12 alkaline extracts. Extracts by other solvents at atmospheric pressure were low either in RG-I pectin yields, RG-I pectin purity or both. Therefore, pH = 13 alkaline at atmospheric pressure appears to be the best options based on yield and purity. However, the design of future RG-I pectin extraction processes should not only include the considerations of novel pectin-based products requirements (e.g. RG-I pectin yield, purity and composition) but also industrial process constraints. For example, water as a solvent is more preferable than strong acid or alkaline in terms of environmental impacts and water extracts do not require any further purification steps to remove the remaining solvent. Atmospheric pressure extractions at 90 °C are safer compared to pressurised hydrothermal extractions, although the optimal MAHE conditions of 130 °C and vapour pressure 2.7 bar are not very high in the context of industrial processes. Furthermore, MAHE has much shorter cycle time of 10 min compared with MAE and CSE of 120 min, and it can also potentially achieve 0 min treatment time, which allows design of a continuous system with a smaller plant footprint.
